# Patch selection by bumble bees navigating discontinuous landscapes

**DOI:** 10.1038/s41598-021-88394-2

**Published:** 2021-04-26

**Authors:** Fabiana P. Fragoso, Qi Jiang, Murray K. Clayton, Johanne Brunet

**Affiliations:** 1 Agricultural Research Service Research Participation Program, Oak Ridge Institute for Science and Education, 455 Science Drive, Madison, WI 53711 USA; 2 Department of Statistics, University of Wisconsin - Madison, 1300 University Ave, Madison, WI 53706 USA; 3 United States Department of Agriculture, Agricultural Research Service, Vegetable Crops Research Unit, 455 Science Drive, Madison, WI 53711 USA; 4 Present Address: Goldman Sachs, 200 West Street, New York, NY 10282 USA

**Keywords:** Ecology, Plant sciences, Zoology

## Abstract

Pollen and nectar resources are unevenly distributed over space and bees must make routing decisions when navigating patchy resources. Determining the patch selection process used by bees is crucial to understanding bee foraging over discontinuous landscapes. To elucidate this process, we developed four distinct probability models of bee movement where the size and the distance to the patch determined the attractiveness of a patch. A field experiment with a center patch and four peripheral patches of two distinct sizes and distances from the center was set up in two configurations. Empirical transition probabilities from the center to each peripheral patch were obtained at two sites and two years. The best model was identified by comparing observed and predicted transition probabilities, where predicted values were obtained by incorporating the spatial dimensions of the field experiment into each model’s mathematical expression. Bumble bees used both patch size and isolation distance when selecting a patch and could assess the total amount of resources available in a patch. Bumble bees prefer large, nearby patches. This information will facilitate the development of a predictive framework to the study of bee movement and of models that predict the movement of genetically engineered pollen in bee-pollinated crops.

## Introduction

Pollen and nectar resources are unevenly distributed over time and space and bees must make many decisions when foraging over the landscape. They must select which patch to visit and choose individual plants, inflorescences and flowers within each patch. Their decision process must remain flexible because resource availability varies both spatially and temporally. Bees often prefer to visit nearby plants and plants that offer constant rewards^1,2^. They use various visual and olfactory cues to select plants^3–5^ and they also learn to associate floral traits and reward^6^. Bees can switch their preferred flower type, for example a flower of certain color or size, based on reward availability^7–9^. In addition, bees prefer inflorescences with larger floral displays^3,7,10,11^. They can detect the number of flowers with pollen or nectar on an inflorescence and thus assess resource availability^7,12^. As bees navigate the landscape to obtain food, their decisions of which plants and inflorescences to visit are strongly influenced by resource availability^7,12^.

While the process used by bees to select plants, inflorescences and flowers has received a lot of attention, less is known about how bees select patches distributed over space. Bees demonstrate patch fidelity^13–15^, which can facilitate pollination of temporally separated flowering species^16^. The total number of flowers they visit in a patch during a foraging bout (residence) is affected by patch size^17^. Previous research of bumble bee foraging at a spatial scale has been guided by the concept of trapline foraging, where bees optimize their travel route over time^18–20^. Bumble bees do not simply move to nearest neighbor flowers but exhibit a tradeoff between minimizing travel distance and favoring more rewarding locations^21–23^. However, most experiments on trapline foraging have been done either in the greenhouse or cages using a few (< 10) artificial flowers or in the wild using a few feeding stations. In general, all flowers or feeding stations present equal reward to the bees. In a typical experiment, bees used the shortest route developed in only 21.4% of all foraging bouts and started using this route after an average of 26.6 foraging bouts^23^. Under these conditions, bees perform 65–80 potential foraging bouts per day and traplining can develop over a matter of hours^18^. Under natural field conditions, however, bees visit patches with many plants and each plant offers a large number of flowers with variable reward. In addition, individual bumble bees (*Bombus impatiens*) in the field make an average of 5.9 foraging trips daily and a foraging trip tends to last over 55 minutes^24^. As a reference point, in a patch of 169 plants, *B. impatiens* individuals were observed visiting, on average, 53.9 flowers in the patch over 3.9 minutes^25^. Bees are therefore likely to visit many patches, plants and flowers during a foraging trip and would need many days to establish a trapline route, assuming it develops after an average of 27 foraging bouts. Moreover, they would need to remember the location of patches, various plants and numerous flowers over many days. While bees learn to quickly optimize a foraging route under controlled conditions, with few flowers or feeding stations presenting similar reward, it is less clear how the concept of trapline foraging can explain bee foraging within and among patches of plants in the field, where each patch contains many plants, each with many inflorescences and flowers offering variable reward. Given these limitations, feeding stations and artificial flowers used in traplining experiments have been equated to patches over the landscape^26^. However, this interpretation assumes that the patch selection process is similar to the process used by bees to select individual flowers. Such an assumption must be tested and it is therefore crucial to examine how bees select patches in a landscape with patchily distributed resources.

**Figure 1:**
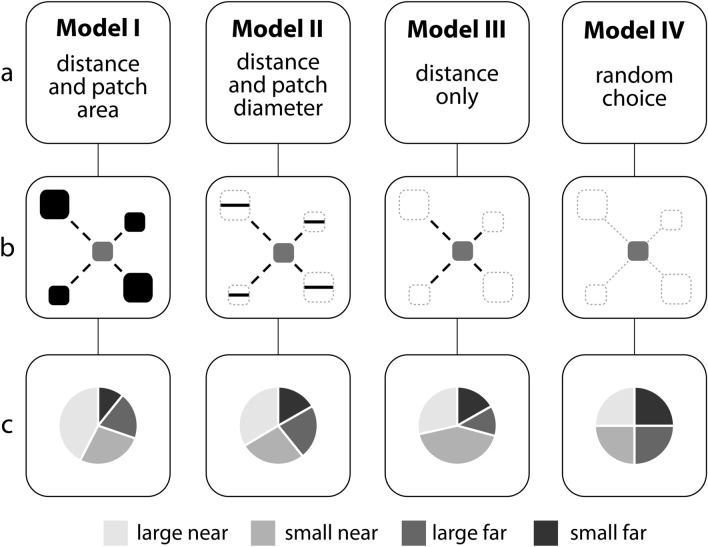
Models of patch attractiveness and derived predictions. (**a**) The parameters of the four models of patch attractiveness. (**b**) Parameters of the four models of patch attractiveness illustrated within the experimental design. (**c**) Predicted probabilities of transitions from the center patch to each of the four peripheral patches derived for each of the four models.

To fill this critical gap in our knowledge of bee foraging behavior, we developed four models of patch attractiveness that differed in how the probability of a bee moving to a patch was influenced by the size of a patch (resources available) and the isolation distance between patches (Fig. 1a). Resource availability influences bee choice of individual plants and inflorescences^7,12^ and minimizing traveling distances over a foraging trip is central to trapline foraging although reward also plays a role^20,22,23,26^. The models also differed in whether bees could estimate resources fully or only partially. Many animals use encounter rate with preys as a measure of resource availability and cannot estimate total resources available^27^. The ability to estimate or not estimate resource availability represents an important assumption of optimal foraging models^28^.

**Figure 2:**
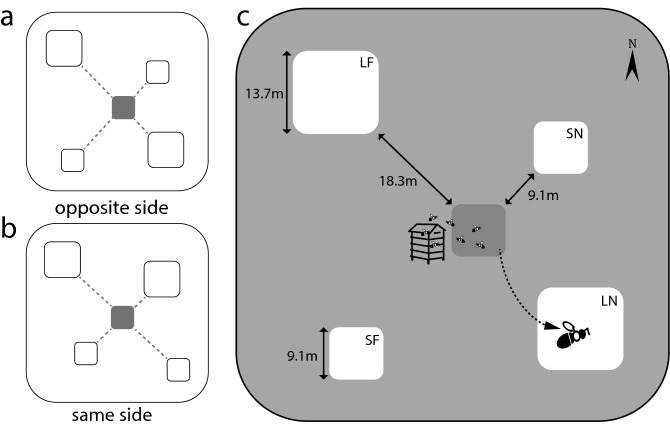
Configurations of the experiment and bee transitions. A center patch surrounded by four peripheral patches of two sizes and at two distances from the center were set up at different sites in two configurations, (**a**) an “opposite side” or (**b**) a “same side” configuration, referring to the location of patches of similar sizes relative to the center patch. (**c**) Bumblebee hives were positioned facing east along the center patch (dark grey) and bee transitions from the center to the peripheral patches (light grey) were recorded over two summers.

To determine which model best described the process by which bumble bees select patches, we set up a field experiment consisting of a center patch and four peripheral patches of two distinct sizes and located at two distances from the center patch (Fig. 1b). The experiment was set up in two configurations with similar size patches on the same or opposite side of the center patch (Fig. 2a, b). Bumble bee hives were placed along one edge of the center patch (Fig. 2c) and empirical transition probabilities from the center to each peripheral patch were obtained over 2 years. Predicted transition probabilities from the center to each of the four peripheral patches were obtained for each of the four models by incorporating the spatial dimensions of a field experiment into the mathematical expression for each model. The best model was selected by comparing observed transition probabilities to the probabilities predicted by each of the four models. By answering how distance and resource availability affect patch selection, and whether bumble bees can estimate resources fully or partially, this study elucidates the decision-making process used by bumble bees when selecting patches. We expect this process to be scale independent and applicable to different plant species. Knowledge gained by this study will advance our understanding of bee foraging behavior over patchily distributed resources and, by doing so, contribute to linking behavioral ecology to landscape ecology^29,30^. In addition, an understanding of the patch selection process used by bees will facilitate the development of models of bee movement over agricultural landscapes that can generate predictions for the movement of genetically engineered pollen in bee-pollinated crops^31^.

## Results

### Predicted values from each model

The first model predicted bees would preferentially move to the large near (LN) patch, followed by small near (SN), large far (LF) and small far (SF) patches (Fig. 1c). A similar trend was predicted for Model II, with smaller probabilities of moving to LN and SN patches and greater probabilities of movement to SF and LF patches, relative to Model I. Model III predicted a greater probability of movement to smaller relative to larger patches. Lastly, Model IV predicted an equal probability of movement to any of the four peripheral patches (Fig. 1c).

### Observed bee transitions

**Figure 3:**
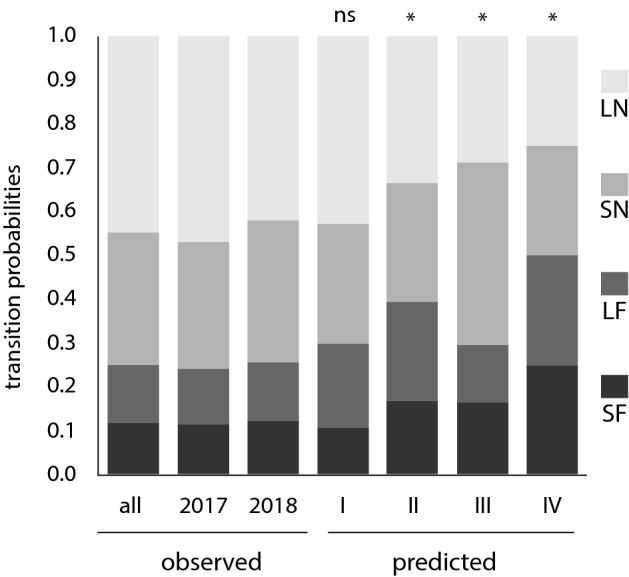
Observed and predicted transition probabilities for each patch type, for both years combined (all), for 2017, and for 2018. Using this figure, one can compare the transition probabilities observed for each patch type, large near (LN), small near (SN), large far (LF), and small far (SF), for a given year (2017 or 2018) or combination of years, to the transition probabilities predicted per patch type by each of the four models of patch attractiveness (I, II, III and IV). We obtained the same pattern for 2017, 2018 and both years combined, with Model I best predicting the observed probabilities of transitioning to the different patch types. A non-significant (ns) model indicates a good fit to the data. A * refers to a model with a probability *P* < 0.05. Model I indicates that bees use information on both distance and total resources available, when selecting a patch.

In total, we recorded 224 transitions from the center patch to one of the four peripheral patches. In 2017, we observed 52 transitions at the site with ‘opposite side’ configuration (Fig. 2a) and 67 transitions at the site with the “same side” configuration (Fig. 2b). In 2018, these numbers were, respectively, 54 and 51 transitions. Observed transition probabilities from the center patch to each of the four peripheral patches (LN, SN, LF and SF) were calculated from these transition data over both years, per year and separately for each site and year (Fig. 3; Table 1).

**Table 1:** Observed transition probabilities for each site each year.

Year	Site	N	LN	SN	LF	SF
2017	M600	67	0.463	0.269	0.134	0.134
2017	B400	52	0.481	0.308	0.115	0.096
2018	M1000	54	0.500	0.148	0.222	0.130
2018	M600	51	0.333	0.510	0.039	0.118

Transition probabilities from the center patch to peripheral patches of two sizes, located at two distances from the center patch with *LN* large near, *SN* small near, *LF* large far and *SF* small far. N is the total number of observed transitions for the site and year.

### Selection of the best model

Model I best explained the observed transition data in all cases examined (Table 2). This means bees are more likely to move to large near patches and can estimate the total amount of resources in a patch. For site M600, Model II could not be rejected in 2017 and Model III in 2018 (Table 2). As discussed in the Methods section for selecting the best model, an outlier was detected for M600 in 2018, and floral density was uneven among peripheral patches at that site that year, with greater density in the small near patch. The fact that a second model, besides Model I, could not be rejected at site M600 each year, suggests an impact of patch configuration on bee movement. However, because Model I consistently explained the observed transition patterns (Table 2), we conclude that bumble bees use information on both distance and total resources available when selecting patches.

**Table 2:** Selection of the best model.

	Model I		Model II		Model III		Model IV	
	*χ*^2^	*P*	*χ*^2^	*P*	*χ*^2^	*P*	*χ*^2^	*P*
Both years	5.85	**0.12**	21.47	< 0.0001	29.54	< 0.0001	65.17	< 0.0001
2017	3.39	**0.33**	13.65	0.003	20.59	0.0001	39.42	< 0.0001
2018	3.13	**0.37**	8.58	0.035	9.69	0.02	26.69	< 0.0001
2017 M600	1.79	**0.62**	6.16	**0.104**	11.17	0.01	19.38	0.0002
2017 B400	2.24	**0.52**	7.98	0.046	9.89	0.02	20.46	0.0001
2018 M600	4.98	**0.083**	6.89	0.032	4.23	**0.120**	14.48	0.0007
2018 M1000	4.24	**0.236**	7.75	0.051	21.88	< 0.0001	19.03	0.0002

Observed transition probabilities were compared against predicted values for each of the four models of patch attractiveness (defined in the text). The Chi-square value and associated probability, *P* value, are presented here. A model with a probability > 0.05 (bolded) indicates a good fit to the empirical data.

## Discussion

Bumble bees consider both patch size and isolation distance when selecting a patch and prefer larger and nearby patches. Visiting large patches provides more resources to bumble bees and flying to the nearest patch minimizes flight energy expenditure. These decisions are likely to optimize the bees net energy intake as optimal foraging theory would predict^28,32,33^. Unlike many animals that use encounter rate with preys as a measure of patch quality and cannot estimate the total resources available^27^, bumble bees possess such an ability. They can estimate the total amount of resources available in the patch.

Although bumble bees might exhibit foraging ranges of hundreds or even thousands of meters^13^ and our inter-patch distances are of a smaller scale than these potential foraging ranges, our experiments are of larger scale than most and we expect the patch selection process identified in this study to be scale independent. In other words, we expect bumble bees to base their decision on distance and total resources available when selecting the next patch, irrespective of the absolute patch sizes. Importantly, we are envisioning patchy resources distributed over the landscape within the foraging range of bees. The equations for the different models can be used to predict transitions between patches of different sizes and distances, so this assumption could be tested in future studies. In addition, we expect the patch selection decision process to be plant species independent and therefore, a similar decision process should apply irrespective of the plant species being examined. Finally, although we concentrated on a single plant species in this study, in order to minimize the number of variables, there are typically patches of different plant species over the landscape and bees may switch plant species while foraging. Bumble bees tend to major on a plant species and can minor on other plant species during a foraging trip^34,35^. With multiple plant species, bees could either use the rules we established in the current study, considering distances and total resources over all patches available, irrespective of the plant species, or they could add an additional rule to decide when to switch to a different plant species. Future studies could determine whether bees apply additional rules to the patch selection process when multiple plant species are available.

Determining the patch selection process used by bumble bees improves our understanding of bee movement and helps translate the study of such movement into a predictive framework^33^. For example, an individual based model describing movement in a continuous landscape, an approach used by Levey and collaborators^36,37^ to examine the effect of corridors on seed dispersal in the eastern blue bird (*Sialia sialis*), can be used to describe bee movement within patches^25^ and be combined with the patch selection process, established here for bumble bees, to describe and predict bee movement in different patchy environments^31^. Because bees may facilitate transgene escape over several kilometers^38^, the large-scale release of genetically engineered crops has created major ecological and economic concerns on the consequences of pollinator-mediated gene flow. There is major interest in modeling the potential spread of pollen from genetically engineered crops into conventional crops^39^ and into populations of cross-compatible weedy or wild relatives^40^. The basic understanding of bee movement patterns provided by this study will facilitate the development of models that predict gene flow for plant species pollinated by bees foraging in patchy environments or discontinuous landscapes. In the United States, the impending release of new varieties produced via gene editing technologies^41^ for various insect-pollinated crops and the new USDA’s plant biotechnology regulations ‘SECURE’ only heighten the criticality of this already imperative issue.

The question remains how bees can estimate distances and the level of resources in a patch. Bees use optic flow—or the retinal image motion experienced during travel—to estimate distance^42,43^. They rely on path integration to keep track of homeward directions by using a sun and polarized-light compass when assessing direction of travel^44,45^ and they can also make use of prominent landmarks to pinpoint their destinations^46^. All these abilities are presumably being employed by bees when estimating distances between patches. Little is known about how bees evaluate total resources available in a patch. The local discontinuity in apparent motion across boundaries allows for flying bees to detect a figure raised from the ground^47^. Pattern and color discrimination, as well as shape detection^48^ would allow bees to evaluate the patch they are heading to. Various visual and potentially olfactory cues^3–5^ are likely being computed by bees to determine the attractiveness of the patch. More work is needed to determine how bees estimate the total amount of resources in a patch but our results indicate that they do not passively visit large patches because they are more apparent, as has been previously suggested^49^.

Landscape configuration, similarly to spatial configuration at smaller scales, can affect bee behavior. Bumble bees have more difficulty making decisions with some spatial configurations than others^18,50^ and our findings indicate that landscape configuration, or the layout of patches over space, may also influence bee behavior. Bees exhibited stronger responses with the opposite side configuration, where only Model I was a good fit to the data. However, the fact that Model I represented a good fit to the data for both field configurations suggest that the patterns of transitions observed are not the results of environmental factors such as wind direction^51^. In addition, we observed variation in the response of bees as some bees did not move to the larger, nearby patch. However, we did not mark individual bees in this experiment and could not follow their decision process over time and thus with experience. A bee may in fact learn the optimal decision with learning and experience^52^ and some bees may learn faster than others reflecting inter-individual differences in how bees handle cognitive tasks and make decisions^53,54^. Future patch selection experiments, marking individual bees in a similar experimental design, would help further clarify the role of learning and experience and individual cognitive differences in the decision-making process of bumble bees selecting patches in a discontinuous landscape.

This study highlights various similarities between the decision-making process bees used to select patches and the one they use to choose inflorescences. When bumble bees visit inflorescences, they prefer inflorescences with larger floral displays^3,10,11^ and base their decision on the number of pollen- or nectar-producing flowers^7,12^. They can estimate the total resources available on inflorescences. Similarly, when visiting patches of plants, bumble bees can estimate the total amount of resources available and prefer patches with more resources. Therefore, bumble bees use a similar criterion, total resources available, when selecting the next inflorescence or the next patch they move to. Moreover, when foraging on inflorescences, bumble bees visit more flowers on larger inflorescences but visit a smaller proportion of the flowers^11,55^. Bumble bees exhibit a similar behavior when foraging on patches of plants where they visit more flowers but a smaller proportion of the flowers in larger patches^17^. With respect to distance, bumble bees prefer nearby patches and seem to prefer nearby plants^1^ although they do not necessarily select nearest neighboring flowers when establishing an optimal trapline^23^. More work is needed to determine the impact of distance and the combined impact of distance and resource availability on inflorescence selection. However, currently available data highlight similarities between the decision-making process of bumble bees when selecting inflorescences and patches of plants. The findings of this study represent an important step towards understanding bee foraging over space, beyond selection of individual flowers or feeding stations.

## Conclusions

Bees consider both distance and patch size and can estimate total resources when selecting patches over the landscape. Moreover, bumble bees follow many similar rules when moving between patches and when selecting inflorescences. This study expands our understanding of bee foraging over patchy resources, a discontinuous landscape, and contributes to a more predictive framework to the study of bee movement. For example, results of this study can facilitate the development of models of the spread of genetically modified pollen in bee-pollinated crops.

## Methods

### Models of patch selection

We contrast four distinct probability models of bee movement between patches which differ in theories of patch attractiveness. In particular, we assume that the probability of a bee moving to one patch—when given the choice of moving to one of several patches—can be described in terms of the size of and the distance to the patch, and that these two factors determine the attractiveness of the patch (Fig. 1a). For mathematical simplicity (and tractability) we assume that a given patch can be represented as a disc of radius *R* whose center is a distance *d* from the pollinator. Moreover, we assume that *d* is much larger than *R*.

Our first two models are the most complex and borrow notions from physics in their construction. In *Model I*, we assume that each point in the patch emits an attractant, whose effect is inversely proportional to the square of the distance from the point to the bee. Suppose that (*x*, *y*) is a point lying on the disc, a distance *r* from the disc center. Then it can be shown that the total attractiveness is given by

$$\iint\limits_{P} {1/\left( {x^{2} + y^{2} + d^{2} } \right) dxdy}$$

where P is the set of points (*x*, *y*) on the disc. By converting to polar coordinates and solving the resultant integral, we see that the total attractiveness is given by

$$2\pi \left( {\ln \left( {1 + \left( \frac{R}{d} \right)^{2} } \right)} \right)$$

Because *d* is assumed to be much larger than *R*, Taylor’s expansion gives the total attractiveness to be proportional to $2\pi \left( \frac{R}{d} \right)^{2}$, or equivalently, the disc Area divided by $d^{2}$. This model incorporates both patch size and isolation distance. It illustrates a case where a pollinator can integrate the area of the patch and determine the total amount of resources available in the patch.

In *Model II*, we assume that the pollinator views the patch (*i.e.* disc) edge-on, and thus only perceives its diameter, and not its area. In that case, the total attractiveness of the patch can be approximated by ${\text{arctan}}\left( \frac{R}{d} \right)$ which, by a Taylor series approximation, can be shown to be proportional to the diameter over distance or 2*R*/*d*. In this model, a pollinator can estimate distance but the probability of choosing a patch is a function of its diameter rather than its area, and therefore the pollinator only obtains partial information about the total resources available.

The next two models are simpler in construction. In *Model III*, we assume that the attractiveness of a patch depends solely on its distance, with the attractiveness being inversely proportional to the squared distance between the pollinator and the center of the patch. This is the closest approximation to a nearest neighbor model except that we are using a more flexible probabilistic approach than the pure nearest neighbor model. Finally, in *Model IV*, we assume that all patches are equally attractive to pollinators, without regard to their size or distance. This model represents a purely random patch choice model.

### Experimental design

We set up an experiment at different sites at the West Madison Agricultural Research Station, WI, USA. The experiment consisted of five patches of alfalfa, a center and two peripheral 9.14 m × 9.14 m patches containing 100 plants (small patches) and two peripheral 13.7 m × 13.7 m patches with 225 plants (large patches). Plants were originally grown from seeds in the greenhouse and transplanted 90 cm apart from each other within each patch. One of the large and one of the small patches were placed 9.14 m diagonally from the center patch (near) while the other large and small patches were placed 18.3 m away (far). This arrangement ensured the presence of four types of peripheral patches: large near (LN), small near (SN), large far (LF) and small far (SF) (Fig. 1b). The spatial configuration of the peripheral patches differed between some sites, with the “opposite side” configuration having same size peripheral patches on opposite sides of the center patch (Fig. 2a) while the “same side” configuration had the two large or two small patches on the same side of the center patch (Fig. 2b). The type of configuration depended on whether similar-size peripheral patches were situated on opposite sides or on the same side of the center patch. The use of different configurations permitted generalization of the results because it minimized potential bias resulting from environmental factors. Tall fescue grass was grown for weed control among patches and among plants within patches and the grass was mowed periodically. At each site, the five alfalfa patches for the experiment were set up within a larger alfalfa hay field which was harvested at least three times throughout the summer prior to flowering. Each year, beehives of the common eastern bumble bee *Bombus impatiens* Cresson, an effective pollinator of alfalfa^56^, were set up along the center patch at each site at least 1 week prior to the beginning of bee observations (Fig. 2c). Observations took place throughout the day, when bees were active.

### Predictions from each of the four models

As noted above, we assume in the mathematical construction that patches are discs to facilitate the necessary calculus. To create a landscape configuration involving patches of different sizes and isolation distances and generate predicted patterns of movement to be tested empirically, we use square patches because these are easier to implement in the field. The mathematically derived attractiveness of a patch above is therefore an approximation to the true attractiveness in the field experiment.

Predictions for the probability of a bee transitioning from the center patch to each of the four conventional patches were made by assigning the dimensions of the field experiment to the variables in the respective equation for each of the four models. Consider our landscape configuration as given in Fig. 2. The areas of the large patches are (13.7 m)^2^ and the areas of the small patches are (9.1 m)^2^; the near and far distances between patches are 9.1 m and 18.3 m, respectively, and the distances from the corners of the patches to their centers are 6.5 m for small patches and 9.7 m for large patches. This allows us to calculate the total distance from the edge of the center patch to the center of each peripheral patch. For the patches abbreviated as LN, SN, LF, and SF for large near, small near, large far and small far, these distances are: 18.8, 15.6, 28.0, and 24.8 m, respectively. Thus, for Model I, the probabilities of movement to the four patches LN, SN, LF, and SF are proportional to 187.69/18.8^2^, 82.81/15.6^2^, 187.69/28.0^2^, and 82.81/24.8^2^, respectively. We therefore calculate those four quantities and multiply by a proportionality constant to ensure that they add to one (because they represent probabilities). Similarly, for the different attractiveness models, we obtained the probabilities of movement to each of the four peripheral patches (Fig. 1c).

### Observed bee transitions

We collected bee transition data over two summers and at two sites each summer. Transition data were collected throughout the day, when bees were active, during the weeks of alfalfa peak bloom. During periods of bee observations, at least two trained observers were positioned on opposite sides of the center patch and followed bumblebees foraging in the patch, especially along the edges. A transition was recorded when a bumblebee was observed to leave the center patch and moved to a peripheral patch and foraged on flowers in that new patch. During the summer of 2017, transition data were collected from site B400 with the ‘opposite side configuration’ and site M600 with the ‘same side’ configuration. In summer 2018, transition data were collected from site M600 and from a new site, M1000, with the opposite side configuration. Site B400 did not have enough plants that survived the winter. Peak flowering at the two sites each year were separated by approximately 2 weeks by mowing the plants at different times in late spring-early summer. Transition data were collected in July for site M600 in 2017 and site M1000 in 2018 and during August for site B400 in 2017 and site M600 in 2018. Observed transition probabilities from the center patch to each of the four peripheral patches were calculated over both years, per year and separately for each site and year. Because *B. impatiens* is the most common bumble bee species in Wisconsin and in the study area, and bees in the experiment were not marked, some of the transitions could result from wild bees and not the bees originating from the hives provided. However, the presence of wild bees would only strengthen the pattern observed in this study because we expect the patch selection decision process of bees examined in this study to be independent of the hive location.

### Selecting the best model

To identify the model that best explained the empirical data, observed transition probabilities were compared to the predictions derived for each of the four models using chi-square goodness of fit tests. For these tests, probability values of *P* ≤ 0.05 indicate that a model is not a good fit to the empirical data. We contrasted observed and predicted transition probabilities over all empirical data (combining sites and years), per year and for each site within each year. All analyses were performed using R 3.5.1 ^57^.

In 2018, patch SN at site M600 had higher floral density relative to the other patches and transition counts were also high. Before performing further analyses for that site and year we first examined whether that patch should be considered an outlier. For the outlier test, a Poisson (generalized linear) regression model was fit whereby we assumed that the transition counts follow a Poisson distribution, and that the log mean of the Poisson distribution could be modeled by a linear combination of “distance” (near or far), “size” (small or large), “year”, “site”, and an indicator variable coding for the observation in question^58^. The resulting *P*-value corresponding to the indicator variable was 0.0016. Multiplying that *P*-value by 4, we had the Bonferroni corrected *P*-value of 0.0064 for testing for an outlier for the M600/2018 site. Given the outlier indicator was statistically significant under these conditions, and given that this patch had a different floral density, we concluded that the high number (*n* = 26) of transitions for the small near patch for site M600 in 2018 could reasonably be considered an outlier. Therefore, to perform the test for site M600 in year 2018 the outlier, patch SN, was excluded and the remaining transition probabilities were scaled up to ensure that they still added to 1.

## Data Availability

The data supporting the findings of this study are available within this paper.
